# Current expert opinion and attitudes to optimize telemedicine and achieve control in patients with asthma in post-pandemic era: The COMETA consensus

**DOI:** 10.1016/j.aprim.2022.102492

**Published:** 2022-10-19

**Authors:** Jesús Molina Paris, Carlos Almonacid Sánchez, Marina Blanco-Aparicio, Javier Domínguez-Ortega, Jordi Giner Donaire, Navidad Sánchez Marcos, Vicente Plaza

**Affiliations:** a Healthcare Center Francia, Fuenlabrada, Madrid, Spain; b Pulmonology Department, Hospital Universitario de Toledo, Toledo, Spain; c Pulmonology Department, Hospital Universitario A Coruña, A Coruña, Spain; d Allergy Department, Hospital La Paz. Institute for Health Research (IdiPAZ), Madrid, Spain; e Pulmonology and Allergy Department, Hospital de la Santa Creu i Sant Pau, Barcelona, Spain; f Spanish Society of Clinical, Family and Community Pharmacy (SEFAC), Madrid, Spain

**Keywords:** Telemedicine, Asthma, Consensus, Delphi methodology, Telemedicina, Asma, Consenso, Metodología Delphi

## Abstract

**Objective:**

To collect perspectives and explore consensus for expert recommendations related to asthma control and the use of telemedicine among professionals who manage patients with asthma.

**Design:**

A Delphi-like questionnaire was designed to analyse the level of agreement about several recommendations formulated by an expert scientific committee about asthma control and the use of telemedicine with this purpose. A dedicated scientific committee validated the questionnaire, which included questions about the participants’ profile and the use of technological tools at a personal level or in clinical practice.

The experts expressed their agreement with a Likert-scale of 9 values: 1–3 was considered no agreement, 4–6 neutral, and 7–9 agreement. A rate ≥70% with the same answer was considered consensus.

**Site:**

The questionnaire was programmed and distributed as an internet-based survey.

**Participants:**

A pre-selected sample of 75 experts with experience in telemedicine (pulmonology, allergology, family medicine, nursing and community pharmacy) responded to a Delphi-like questionnaire composed by six questions and 52 items.

**Interventions:**

Consultation was performed in two consecutive waves: the first wave was carried out from 12th of July to 8th of September of 2021; the second wave, from 25th of October to 12th of November of 2021.

**Main measurements:**

Three questions about asthma control (actions for achieving or maintaining control of asthma at every visit, current problems that affect asthma control, and potential solutions to offset such problems), and three questions about the impact of telemedicine in asthma control (potential benefits of telemedicine, and potential reticence about telemedicine among both patients and healthcare professionals) were included.

**Results:**

From the 52 items inquired, 35 were agreed by consensus. The actions for achieving or maintaining control of asthma, the problems that affect asthma control, and their potential solutions were agreed by consensus. The potential benefits of telemedicine were validated by consensus. None of the potential reservations of patients about telemedicine were validated, while five out of 14 potential reservations of healthcare professionals were agreed by consensus.

**Conclusions:**

The COMETA consensus provides a current picture of the main problems for achieving asthma control, the benefits and the reservations about the use of telemedicine in the Spanish setting, and offers solutions. A wide interest in implementing telemedicine has been observed, although current limitations need to be overcome.

## Abstract (es)

**Objetivos:**

Recoger las perspectivas y explorar el consenso de los expertos en las recomendaciones para un mejor control del asma y el uso de la telemedicina entre los profesionales que tratan pacientes con asma.

**Diseño:**

Se diseñó un cuestionario con la metodología Delphi para analizar el nivel de acuerdo en varias recomendaciones formuladas por un comité científico experto sobre el asma y el uso de la telemedicina. Un comité experto validó el cuestionario, que incluyó preguntas sobre el perfil de los participantes y el uso de las herramientas tecnológicas a nivel personal y en la práctica clínica.

Los expertos expresaron su acuerdo con una escala de Likert de 9 valores: 1-3 se consideró sin acuerdo, 4-6 neutral y 7-9 de acuerdo. Se consideró consenso cuando ≥70% de los participantes respondieron la misma respuesta.

**Ubicación:**

La consulta se realizó online.

**Participantes:**

Una muestra preseleccionada de 75 expertos con experiencia en telemedicina (neumología, alergología, medicina familiar, enfermería y farmacia comunitaria) respondió a un cuestionario formado por 6 preguntas y 52 ítems.

**Intervenciones:**

La consulta se realizó en dos olas consecutivas: la primera ola tuvo lugar desde el 12 de julio al 8 de septiembre de 2021. Y la segunda ola, del 25 de octubre al 12 de noviembre de 2021.

**Medidas principales:**

En el cuestionario se incluyeron tres preguntas sobre el control del asma (acciones para lograr y/o mantener el control del asma, problemas actuales que afectan a este control y las posibles soluciones), y tres preguntas sobre el impacto de la telemedicina en el control del asma (potenciales beneficios de la telemedicina y la posible reticencia a telemedicina entre los pacientes y los profesionales sanitarios).

**Resultados:**

De los 52 ítems consultados, en 35 de ellos se alcanzó el consenso. Se acordaron por consenso las acciones para lograr o mantener el control del asma, los problemas que afectan al control del asma y sus posibles soluciones. Los beneficios potenciales de la telemedicina fueron validados por consenso. Ninguna de las posibles reservas de los pacientes sobre la telemedicina fue validada, mientras que cinco de las 14 posibles reservas de los profesionales sanitarios se acordaron por consenso.

**Conclusiones:**

El consenso COMETA ofrece una imagen actual de los principales problemas para lograr el control del asma, los beneficios y las reservas sobre el uso de la telemedicina en el ámbito español, y ofrece soluciones. Se ha observado un amplio interés por parte de los profesionales sanitarios por implementar la telemedicina, aunque es necesario superar las limitaciones actuales.

## Introduction

Asthma is a common chronic inflammatory respiratory disease that affects 5% of adult population and 10% of paediatric population in Spain.^1^

Asthma control is achieved when therapeutic interventions foster the absence or maximum reduction of symptoms.^2^, ^3^ The concept of control encompasses both the current control of symptoms and the future risk of adverse outcomes.^4^, ^5^ The adequate control of asthma is usually compromised and a considerable percentage of patients tend to present acute exacerbations, visit the emergency department, or even be admitted in hospitals.^6^

COVID-19 pandemic has entailed, among other consequences, consecutive lockdowns of the population, limitations of healthcare human resources, and a loss of face-to-face follow-up of patients with chronic diseases, events that could contribute to a worse control of patients with asthma. Although asthma does not seem to be associated with a higher risk of acquiring or presenting severe COVID-19 disease,^7^, ^8^ it has been described that morbidity or some medications lead to poorer clinical outcomes.^8^, ^9^, ^10^ Therefore, it is important to ensure a good control of symptoms to improve the management of patients with asthma.

In this new pandemic frame, many new strategies have been proposed and implemented with the purpose of ameliorating care of patients with asthma. Telemedicine has shown to improve the control of asthma symptoms, the quality of life (QoL), and some patient-reported outcomes of patients with asthma.^11^, ^12^, ^13^ Likewise, the impact of some eHealth interventions has been positive in terms of adherence to inhalers, avoidance of rescue medication, and satisfaction of patients.^14^, ^15^

In light of the current situation, the COMETA (Spanish acronym for *Control as Goal in the Era of Telemedicine in Asthma*) project emerged to promote the use of telemedicine with asthma control purposes.^16^ In a first step, a dedicated multidisciplinary scientific committee identified existing problems for achieving a proper control of asthma, and proposed initiatives for mitigating them, telemedicine-based strategies, and an algorithm for teleconsultation.^17^ In a second step, the current study was performed with the aim to collect information and explore consensus about some expert recommendations related to asthma control and the use of telemedicine among professionals involved in the management of patients with asthma.

## Participants and methods

### Design

A Delphi-like questionnaire was designed to analyse the level of agreement about several recommendations formulated by an expert scientific committee about asthma control and the use of telemedicine with this purpose. This method allows a series of successive surveys with a feedback loop that ease the revision of the responses of the panel.^18^

A dedicated scientific committee validated the questionnaire, which included questions about the participants’ profile and the use of technological tools at a personal level or in clinical practice. Three questions about asthma control (actions for achieving or maintaining control of asthma at every visit, current problems that affect asthma control, and potential solutions to offset such problems), and three questions about the impact of telemedicine in asthma control (potential benefits of telemedicine, and potential reticence about telemedicine among both patients and healthcare professionals) were included. The questionnaire was programmed and distributed as an internet-based survey.

Consultation was performed in two consecutive waves: the first wave was carried out from 12th of July to 8th of September of 2021; the second wave, from 25th of October to 12th of November of 2021.

### Sample and participants

A pre-selected multidisciplinary panel composed by 75 experts in pulmonology, allergology, family medicine, nursing and community pharmacy participated in the survey. The experts had to fulfil all the pre-established inclusion criteria: (a) having more than 5 years of experience with patients with asthma; (b) having used telemedicine within the last six months; and (c) belonging to some respiratory disease task force for family medicine physicians and nurses, or to an asthma task force for community pharmacists.

### Analysis

The experts expressed their agreement with each item included in the questions with a Likert scale of 9 values, being 1 the lowest agreement and 9 the highest. For interpretative purposes, 1–3 was considered lack of agreement; 4–6, neutral; and 7–9, agreement. For the question about the use of technological tools at a personal/professional level, a Likert scale of 10 values was used: 1–2 were considered no use; 3–4, little use; 5–6, occasional use; 7–8, regular use; and 9–10 constant use. Results are shown as the percentage of participants who agreed with the same answer. Consensus was considered when 70% of participants or more expressed the same answer. During the first wave, for questions 12 and 16, participants were asked to propose answers other than those suggested, and they were categorized during the second wave to select the most popular options. A descriptive statistical analysis was performed for all variables. Continuous variables were summarized by mean value and standard deviation (SD). Categorical variables were described by number of cases and percentage.

**Figure:**
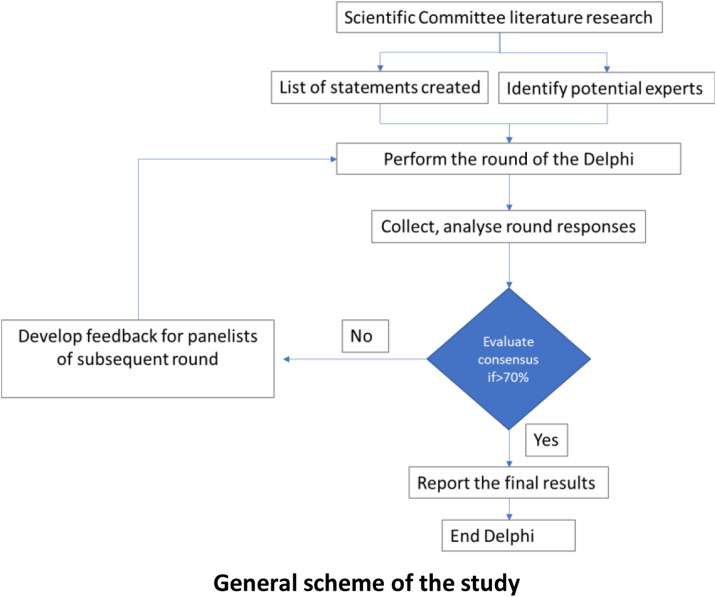

## Results

### Characteristics of participants

All the experts invited answered both waves, and the percentage of participation was 100%. Mean age of participants was 50.3 years (SD 9.1). Mean years of experience was 22 (SD 9.1). Mean number of patients with asthma visited per month was 58 (SD 57.1): 26.7 (SD 18.4) with mild asthma, 29.2 (SD 16.8) with moderate asthma, 25.8 (SD 25.9) with severe asthma, and 18.3 (SD 16.9) with intermittent asthma. Distribution by gender, specialties, type of site, and years of experience is specified in Table 1.

**Table 1:** Characteristics of participants.

Characteristics	No. of participants (%) (*n* = 75)
*Gender*	
Men	33 (44.0%)
Women	42 (56.0%)

*Specialties*	
Allergologists	18 (24.0%)
Pulmonologists	18 (24.0%)
Family medicine physicians	18 (24.0%)
Community pharmacists	10 (13.3%)
Nurses specialised in family medicine	5 (6.7%)
Nurses specialised in allergology	3 (4.0%)
Nurses specialised in pulmonology	3 (4.0%)

*Entitlement of working centre*	
Public	73 (97.3%)
Mixed	2 (2.7%)
Private	0 (0.0%)

*Type of working centre*	
Primary care	33 (44.0%)
Third level hospital	34 (45.3%)
Second level hospital	6 (8.0%)
First level hospital	2 (2.7%)

*Years of experience*	
>16 years	53 (70.7%)
11–15 years	13 (17.3%)
8–10 years	5 (6.6%)
≤7 years	4 (5.3%)

*Number of patients with asthma visited per month*	
≥41	35 (46.7%)
31–40	6 (8.0%)
21–30	8 (10.7%)
11–20	14 (18.7%)
≤10	12 (16.0%)

### Use of telemedicine among participants

**Figure 1:**
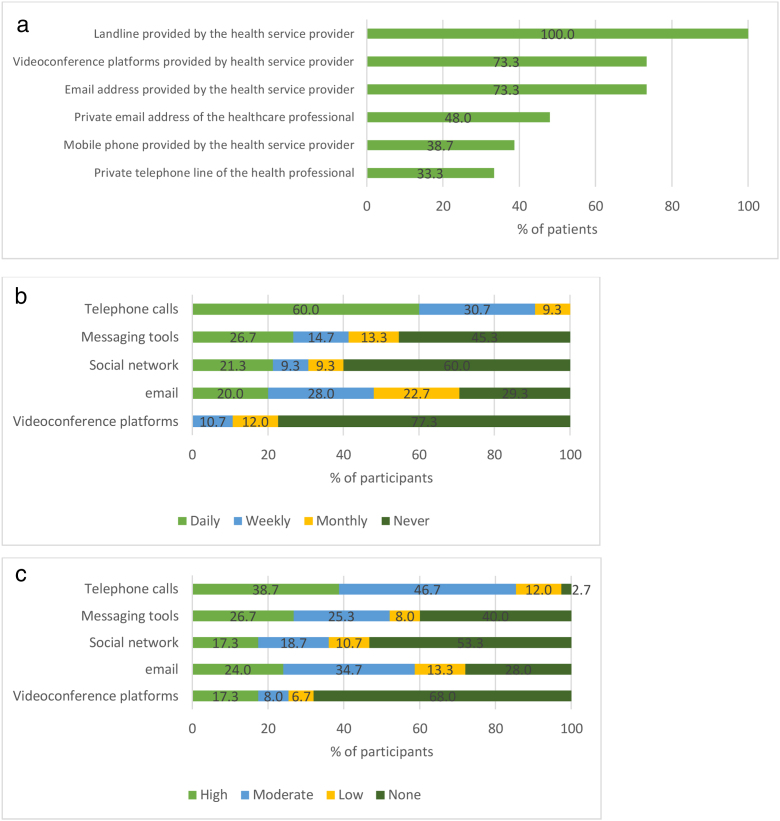
Telemedicine tools: (a) current resources of the participants; (b) frequency of use of telemedicine tools among participants; (c) satisfaction with telemedicine tools among participants.

A total of 96% of participants answered they will still use telemedicine for managing their patients after COVID-19 pandemic. Landline, videoconference platform, email address and mobile phone were provided by health services providers for, respectively, 100%, 73.3%, 73.3% and 38.7% of the participants. Respectively, 48% and 33.3% of participants declared to still use their private e-mail address and their private telephone line for work purposes (Fig. 1a).

**Figure 2:**
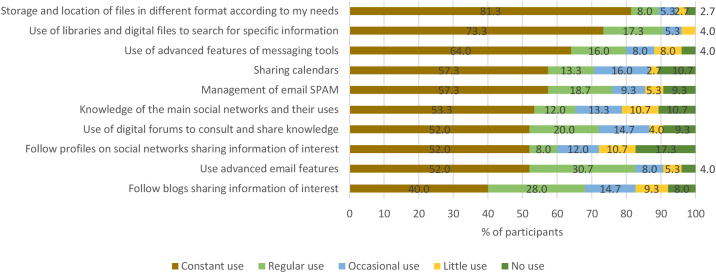
Use of technological tools at a professional and/or personal level.

The frequency of use of different telemedicine tools on a weekly or daily basis was the following: 90.7% of participants used telephone calls, 48.0% used email, 41.3% used messaging tools, 30.7% used their social networks; and 10.7% used videoconference platforms (Fig. 1b). Telephone calls were especially common among family medicine physicians and nurses specialised in allergology and pulmonology, as 100% of them declared to perform daily or weekly calls. Messaging tools were more popular among community pharmacists and nurses specialised in allergology and pulmonology, as more than 60% of them used it on a daily or weekly basis. The percentage of participants expressing high-moderate satisfaction with the use telephone calls was 85.3%, with messaging tools 52.0%, with email 58.7%, with social network 36.0%, and with videoconference platforms 25.3% (Fig. 1c). Storage and location of files in different format, the use of advanced features of messaging tools, the use of libraries and digital files and the use advanced email features were the technological tools constantly or regularly used by more than 80% of participants (Fig. 2).

Participants were invited to suggest aspects that could improve regarding the use of telemedicine in their clinical practice, and they included the following: knowledge and training on useful apps; knowledge about legal protection; better internal communication in primary care and multidisciplinary teams; renewal of equipment, software, network, landlines and other technologic resources; increased use of videoconference platforms; greater collaboration of patients; planification and integration of telemedicine outcomes; and modification of policies to develop real telemedicine and not just teleadministration.

### Control of asthma

**Figure 3:**
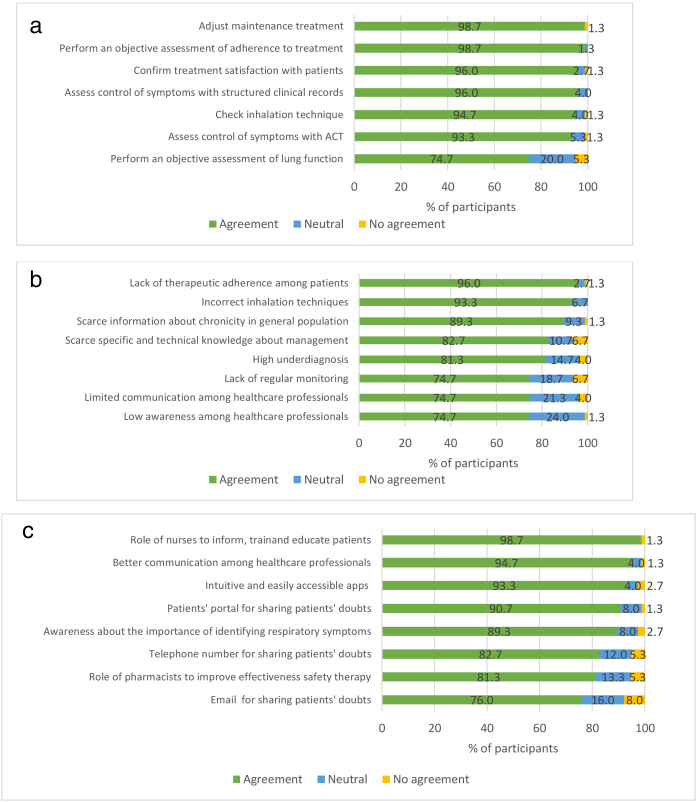
Level of consensus in the questions related to the control of the symptoms of asthma: (a) actions to be taken at each visit to reach or maintain the control of asthma; (b) problems that currently affect the control of asthma; (c) solutions for those problems that currently affect the control of asthma.

All the proposed actions for reaching or maintaining asthma control that can be taken during medical visits were agreed by consensus. The most rated actions were the adjustment of maintenance treatment and the objective assessment of adherence at each visit, both agreed by 98.7% of participants (Fig. 3a).

All the problems currently affecting asthma control presented in the survey were agreed by consensus. The lack of therapeutic adherence (96.0%), followed by the inhalation technique-related mistakes (93.3%), were the items agreed as problems by more participants (Fig. 3b).

All the items proposed as solutions for the current lack of asthma control in the survey were agreed by consensus (Fig. 3c). The most rated were the role of nurses in information, training and education (98.7%), the better communication among professionals (94.7%), and the use of intuitive and accessible apps (93.3%). Participants were asked to propose other solutions than those included in the questionnaire, from which the following were prioritized: the configuration of national plans or strategies for the management of asthma; self-management programmes for patients; programmes for telemedicine access; promotion of respiratory diseases in the portfolio of services; electronic alert systems for prescription refill; educational projects and resources for professionals to promote comprehensive responses, to motivate behavioural changes among patients and to prescribe a correct treatment for asthma; and protocolized implementation of patient-professional communication channels.

### Telemedicine

**Figure 4:**
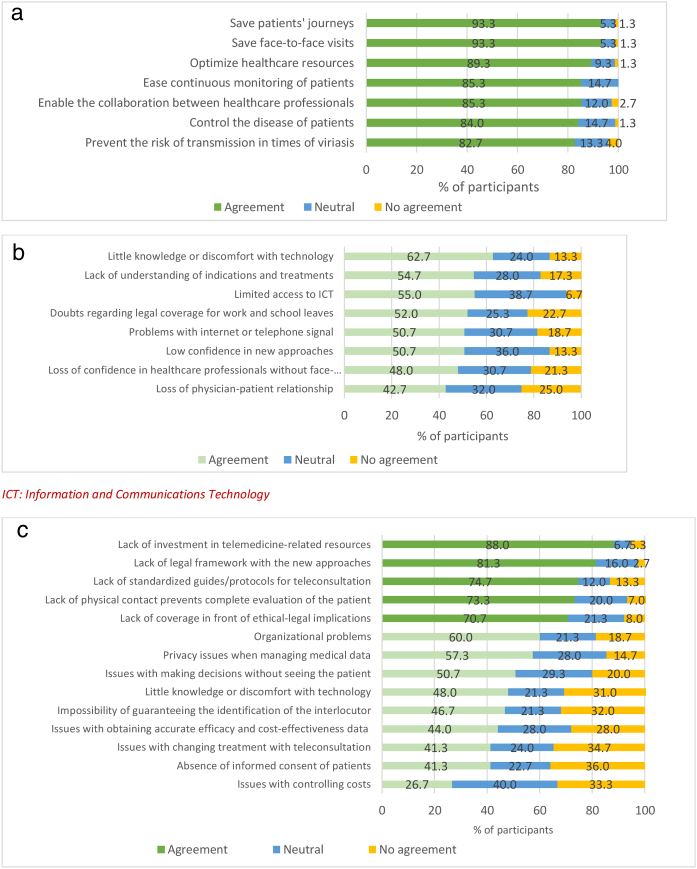
Level of consensus in the questions related to the use of telemedicine for patients with asthma: (a) benefits of telemedicine among patients with asthma; (b) reticence about telemedicine among patients; (c) reticence about telemedicine among healthcare professionals. ICT: Information and Communications Technology.

More than 80% of the participants agreed to the seven items proposed as benefits of telemedicine among patients with asthma. Time and cost savings on both patients’ journeys and face-to-face visits were the most appreciated benefits, considered as such by 93.3% of the sample (Fig. 4a). Considering that this survey was answered in times of COVID-19 pandemic, the prevention of the risk of viral transmission was also rated as an important benefit by 82.7% of participants. Other potential benefits of telemedicine for patients suffering from asthma were proposed by the participants, and the following were prioritized: possibility of education and checking of the inhalation technique; immediate detection and resolution of patients’ unmet needs or doubts; shortening of diagnosis and treatment adjustment time; greater approach to young patients; economic savings; improved pharmacist-patient communication; creation of virtual groups of patients; personalization of healthcare; higher perception of self-control and follow-up; and collection of objective measures (questionnaires or spirometry).

Physicians were enquired about the reservations that either patients or healthcare professionals usually present regarding the use of telemedicine. None of the eight items proposed as reservations of patients with telemedicine were agreed by consensus (Fig. 4b). In fact, less than 50% of participants considered the loss of confidence in healthcare professionals and the loss of physician-patient relationship as relevant. Conversely, five out of the 14 items suggested as possible reservations of healthcare professionals were agreed by consensus (Fig. 4c): the lack of investment in telemedicine resources (88.0%), of a legal framework with the new approaches (81.3%), of standardized guides/protocols for teleconsultation (74.7%), of physical contact (73.3%), and of ethical-legal coverage (70.7%). Other reservations from healthcare professionals were proposed and rated, and the following were the most important: obsolescence of current equipment and software; lack of human, material and technical resources; lack of standardized telemedicine platforms; impact on equity due to the exclusion of patients with technological difficulties; impossibility to see patients; loss of non-verbal communication; inability to teach or check the inhalation technique; email needed to send instructions, questionnaires and reports to the patient; higher number of missed appointments; and lack of response of patients to calls.

## Discussion

The COMETA consensus has shown a high level of agreement among a wide sample of experts on the evaluation of several recommendations related to asthma control and the use of telemedicine that had been previously agreed by a reduced multidisciplinary group.^17^ Consequently, the implementation of all the actions to be taken to reach asthma control and the solutions for the issues that currently affect the control of asthma that have been stated in this work may help to improve asthma control through teleassistance.

The current work shows that asthma control is a fundamental problem in the management of patients. In a study with a wide sample of patients 45% of patients remained uncontrolled, 44% had used oral corticosteroids in the previous 12 months, 24% had visited an emergency department, and 12% had been hospitalised, although most of them regarded their asthma as controlled.^6^ Thus, achieving a good control is one of the main current goals of healthcare professionals managing patients with asthma.^19^ This study found that many actions in virtual care such as the promotion of nurses’ role, the improvement of communication among healthcare professionals, and the use of apps or patients’ portals for sharing doubts can help achieve asthma control.

Some of the already known potential problems to achieve a good control of asthma have been validated in this study: lack of adherence, incorrect inhalation techniques, lack of knowledge about the pathology, underdiagnoses and lack of regular monitoring, among others. In light of this, participants agreed on a pool of actions that should be taken at every visit to reach or maintain asthma control: the assessment of lung function and symptoms, the inhalation technique, and the satisfaction with inhalation devices, as they exert an impact on asthma control^19^, ^20^ and many strategies have been proposed to achieve effective use of inhalers.^21^

Since the onset of telemedicine, many publications position it as a useful and effective tool for the management of patients with asthma,^22^, ^23^, ^24^ for the empowerment of patients,^25^, ^26^ and for reducing costs.^27^ Telemedicine might also help to counteract some of the essential problems to achieve asthma control nowadays, although face-to-face visits will continue to be essential. The participating experts showed a good predisposition to incorporate telemedicine in their clinical practice, although resources provided by healthcare providers seemed to be insufficient in some cases: a high percentage of professionals were still using their private email accounts or telephone line for work purposes, and videoconference platforms were not always available. Not even half of the sample declared to use email or messaging tools on a weekly or daily basis, and only one out of ten did so with videoconference platforms.

There was wide consensus about the benefits brought by telemedicine (saving and optimization of resources of both patients and healthcare system, continuous monitoring and control of patients and better multidisciplinary communication), but not about the reservations that patients and professionals present regarding telemedicine. The only agreed items were related to the lack of investment, legal framework and coverage, and protocols and guidelines, and with the consequences of a poor physical contact with patients.

## Limitations

The design of this study presents some limitations intrinsic to the chosen methodology. The sample was pre-selected and might not be representative of the population of healthcare professionals managing patients with asthma. However, the purpose was to count on a sample of professionals with high degree of experience and acquainted with telemedicine, rather than a randomized sample. Moreover, the selected sample allowed a response rate of 100%. Additionally, this study did not consider patients’ participation, so the information collected regarding patients’ potential attitudes towards telemedicine rely on physicians’ perspective.

The number of participants of certain specialties was lower than others. Once again, our objective was not to compare results among specialties, but rather to obtain the opinion among physicians and other professionals participating in the management of the patient.

The Delphi method is carried out with pre-defined questions and answers, which limit the contribution of personal ideas or clarifications. In order to counteract this limitation, the questionnaire was provided with spaces to fill with personal contributions. Moreover, panellists were asked to propose answers other than those suggested in several questions: such as the aspects to improve in the use of telemedicine in clinical practice, the problems affecting the control of asthma, the potential benefits of telemedicine, and the reticence with telemedicine of healthcare professionals.

## Conclusions

The COMETA consensus provides a current picture of the follow-up that patients with asthma need in order to achieve control, through the main problems and solutions for better asthma control. Likewise, it offers the perception of healthcare professionals regarding the use of telemedicine through the evaluation of its benefits and the reservations of both professionals and patients. According to the results obtained, there is a wide interest in implementing telemedicine in daily clinical practice, but it is necessary to work on the limitations.

**What is known on the subject**

• The adequate control of asthma is a fundamental problem in the management of patients, and it is usually compromised. In European countries rates of uncontrolled patients, use of corticosteroids in the previous 12 months, emergency department visits and hospitalizations remain high although most patients regard their asthma as controlled.
• The consequences of COVID-19 pandemic could contribute to a worse control of patients with asthma.
• Achieving a good control is one of the main current goals of healthcare professionals managing patients with asthma.

**What this study contributes**

• The participants of this study agreed about the potential problems to achieve good asthma control, mainly related to adherence and inhalation technique, and actions to overcome them, such as promoting the role of nurses in information, training and education or encouraging a better communication among healthcare professionals.
• The adjustment of maintenance treatment; the objective assessment of adherence, symptoms, and inhalation technique; and the confirmation of satisfaction are actions agreed to be taken at each visit to reach or maintain the control of asthma.
• The participants of this study agreed about the benefits of telemedicine, including the saving and optimization of patients and healthcare system resources, the continuous monitoring and control of patients, and the multidisciplinary communication. There is a wide interest in implementing telemedicine in daily clinical practice, but some limitations need to be overcome.

## Funding

Nothing to declare.

## Ethical considerations

Nothing to declare.

## Conflict of interests

COMETA is a project supported by GSK and developed by independent scientific committee formed by the authors of the manuscript.

J.M. declares that he has received fees from AstraZeneca, Boehringer Ingelheim, GSK, Menarini, Novartis, Orion Pharma, Pfizer, semFYC, SOMAMFYC and SERMAS for participation in meetings, consultancies or conferences.

C.A. declares having received fees from ALK, AstraZeneca, Chiesi, GSK and Novartis for his participation in meetings, consultancies, conferences or research.

M.B. declares having received fees from ALK, AstraZeneca, Chiesi, GSK, Novartis, Teva and Zambon for her participation in meetings, consultancies, congresses or research.

J.D. declares having received fees fromALK, AstraZeneca, Bial, Cjiesi, GSK, Leti Pharma, Menarini, Mundipharma, Novartis, Sanofi and TEVA for his participation in conferences or consultancies.

J.G. declares having received fees from AstraZeneca, Boehringer Ingelheim, GSK, Mundipharma, Menarini and Pfizer for his participation in meetings, consultancies, conferences or research.

N.S. declares having received fees from GSK, Pfizer, Sanofi, Teva and Zambon for his participation in meetings, consultancies or conferences.

V.P. declares having received fees from ALK, AstraZeneca, Boehringer Ingelheim, Chiesi, GSK, Menarini and Sanofi for his participation in meetings, consultancies, conferences or research.
